# Personalized nutrition in type 2 diabetes remission: application of digital twin technology for predictive glycemic control

**DOI:** 10.3389/fendo.2024.1485464

**Published:** 2024-11-20

**Authors:** Paramesh Shamanna, Shashank Joshi, Mohamed Thajudeen, Lisa Shah, Terrence Poon, Maluk Mohamed, Jahangir Mohammed

**Affiliations:** ^1^ Bangalore Diabetes Center, Bangalore, Karnataka, India; ^2^ Department of Diabetology and Endocrinology, Lilavati Hospital and Research Center, Mumbai, India; ^3^ Twin Health, Mountain View, CA, United States

**Keywords:** digital twin technology, personalized nutrition, type 2 diabetes remission, predictive glycemic control, machine learning

## Abstract

**Background:**

Type 2 Diabetes (T2D) is a complex condition marked by insulin resistance and beta-cell dysfunction. Traditional dietary interventions, such as low-calorie or low-carbohydrate diets, typically overlook individual variability in postprandial glycemic responses (PPGRs), which can lead to suboptimal management of the disease. Recent advancements suggest that personalized nutrition, tailored to individual metabolic profiles, may enhance the effectiveness of T2D management.

**Objective:**

This study aims to present the development and application of a Digital Twin (DT) technology—a machine learning (ML)-powered platform designed to predict and modulate PPGRs in T2D patients. By integrating continuous glucose monitoring (CGM), dietary data, and other physiological inputs, the DT provides individualized dietary recommendations to improve insulin sensitivity, reduce hyperinsulinemia, and support the remission of T2D.

**Methods:**

We developed a sophisticated DT platform that synthesizes real-time data from CGM, dietary logs, and other biometric inputs to create personalized metabolic models for T2D patients. The intervention is delivered via a mobile application, which dynamically adjusts dietary recommendations based on predicted PPGRs. This methodology is validated through a randomized controlled trial (RCT) assessing its impact on various metabolic markers, including HbA1c, metabolic-associated fatty liver disease (MAFLD), blood pressure, body weight, ASCVD risk, albuminuria, and diabetic retinopathy.

**Results:**

Preliminary data from the ongoing RCT and real-world study demonstrate the DT’s capacity to generate significant improvements in glycemic control and metabolic health. The DT-driven personalized nutrition plan has been associated with reductions in HbA1c, enhanced beta-cell function, and normalization of hyperinsulinemia, supporting sustained T2D remission. Additionally, the DT’s predictions have contributed to improvements in MAFLD markers, blood pressure, and cardiovascular risk factors, highlighting its potential as a comprehensive management tool.

**Conclusion:**

The DT technology represents a novel and scalable approach to personalized nutrition in T2D management. By addressing individual variability in PPGRs, this method offers a promising alternative to conventional dietary interventions, with the potential to improve long-term outcomes and reduce the global burden of T2D.

## Introduction

Type 2 Diabetes (T2D) is a complex metabolic disorder characterized by chronic hyperglycemia, driven by a combination of insulin resistance and impaired beta-cell function (1). The global rise in T2D prevalence has resulted in significant healthcare burdens, particularly due to complications such as cardiovascular disease, nephropathy, and neuropathy (2). Traditional management approaches, including pharmacotherapy and standardized nutritional guidelines, often fail to achieve long-term glycemic control and remission. This shortcoming is largely due to the inability of these approaches to address the significant interindividual variability in metabolic responses (2).

Conventional “one-size-fits-all” nutritional strategies are typically based on population averages, which often disregard the unique physiological and metabolic profiles of individual patients (3). This can lead to suboptimal outcomes, even with tightly controlled lifestyle interventions, as results can vary widely across different individuals (4). This variability is particularly evident in postprandial glycemic responses (PPGRs), where significant differences are observed among individuals consuming the same meal (5).

Low-calorie and low-carbohydrate diets are common approaches in T2D management, but both have limitations. Calorie-restricted diets, such as those in the Diabetes Remission Clinical Trial (DiRECT) trial, reduce hyperinsulinemia and hepatic fat, improving insulin resistance and lowering glucose output (6). However, these diets can risk causing protein malnutrition, micronutrient deficiencies (7), and essential fatty acid shortages, potentially leading to adverse outcomes such as cardiac arrhythmias (8), increased mortality, and rebound weight gain, which may trigger T2D relapse after remission (9). Similarly, low-carbohydrate diets, which focus on reducing macronutrient intake, can achieve similar results by lowering glucose spikes, reducing glucolipotoxicity, and enhancing beta-cell function. However, they often overlook other critical factors that influence PPGRs and fail to account for individual variability, resulting in low remission rates, typically around 4-10% after one year (10).

The limitations of these conventional approaches highlight the need for a more personalized strategy in managing T2D. Recent advancements in personalized nutrition propose a novel approach to managing T2D by tailoring dietary recommendations based on individual metabolic characteristics. Personalized nutrition considers factors such as genetics, gut microbiome composition, habitual diet, and lifestyle elements like physical activity and sleep patterns. Machine learning (ML) algorithms used to predict PPGRs based on dietary, anthropometric, physical activity, and gut microbiota data have demonstrated promising accuracy, with validation in independent cohorts. However, most data-driven approaches, including metabolic phenotyping and predictive model development, have primarily focused on non-diabetic and prediabetic populations. These models have yet to fully address the complexities of predicting PPGRs in individuals with T2D, who experience greater glycemic variability and often use glucose-lowering medications, complicating predictions compared to non-diabetic populations (11).

Recent studies have revealed significant variability in PPGRs among individuals consuming the same standardized meal. The linear regression between PPGRs and carbohydrate content was positive for nearly all participants (95.1%), indicating higher PPGRs with carbohydrate-rich meals. However, the degree of carbohydrate sensitivity varied widely, reflecting the broad spectrum of glycemic responses among individuals, independent of carbohydrate consumption levels (12).

The Personalized Responses to Dietary Composition Trial 1 (PREDICT 1) clinical trial quantified and predicted individual variations in postprandial triglyceride, glucose, and insulin responses to standardized meals (13). The study revealed significant variability, even among identical twins, driven largely by modifiable factors. For instance, gut microbiome composition accounted for a considerable portion of these variations, underscoring the importance of non-genetic influences on metabolic responses. Additionally, factors such as meal timing, sleep, and physical activity were found to be particularly predictive of postprandial responses, while genetics played a less significant role than previously believed. ML models that incorporated these variables accurately predicted individual postprandial triglyceride and glycemic responses, with consistent validation results in a U.S. cohort. These findings challenge the effectiveness of standardized dietary recommendations and support the potential of personalized nutrition for broader disease prevention. However, the PREDICT 1 study had limitations, particularly when compared to findings from the Israeli cohort (11). The correlation of intra-individual PPGRs in PREDICT 1 was lower, possibly due to the choice of a standardized meal (bagel and cream cheese), which may not have elicited consistent glycemic responses compared to meals used in the Israeli cohort. Additionally, PPGR prediction accuracy in the PREDICT 1 cohort was slightly lower, suggesting that meal composition and other population-specific factors may have affected model performance (13).

Among European adults, personalized nutrition advice delivered via the internet produced larger and more appropriate changes in dietary behavior than conventional approaches. However, the online nature of this study limited the range of measures, with some key health biomarkers, like blood pressure, not recorded. Data were self-reported or collected remotely, introducing potential measurement errors (14).

In response to the limitations of conventional strategies, we developed and implemented Digital Twin (DT) technology (15–17). This advanced approach addresses the shortcomings of previous models (11, 13) by offering a more personalized method for predicting PPGRs in individuals with T2D. Unlike models focused on non-diabetic or prediabetic populations (11), DT technology integrates continuous glucose monitoring (CGM) data, medication use, dietary logs, physical activity, and other physiological metrics specific to T2D patients. This allows the DT model to account for the greater glycemic variability and complex medication regimens typical in these individuals. The DT approach represents a significant advancement in T2D management by creating a comprehensive, personalized intervention that addresses the multifaceted nature of the disease (18).

By combining CGM data, dietary logs, physical activity, and other inputs, the DT system creates a dynamic, individualized model of a patient’s metabolism. Using machine learning algorithms, it predicts personalized PPGRs and generates real-time dietary recommendations to minimize glucose fluctuations, thereby optimizing glycemic control and potentially enabling T2D remission. The predictive accuracy of DT technology has been validated in a randomized controlled trial (RCT) (18), showing significant improvements in both glycemic control and personalized dietary recommendations. These DT-driven interventions allowed for more precise, real-time dietary adjustments, leading to better overall metabolic health and increased rates of T2D remission.

This personalized approach, accessible through mobile and web applications, enables real-time assessments of how specific foods affect blood glucose levels. It offers benefits comparable to low-calorie or low-carbohydrate diets but without the need for extreme dietary restrictions. Instead, patients can achieve similar results—such as reducing hepatic and pancreatic fat, improving insulin resistance, and promoting remission—using home-cooked meals rather than meal replacements (18).

Clinical application of DT technology has shown significant promise in managing T2D. Patients following the DT-guided personalized nutrition plan have experienced notable improvements in glycemic control, with many achieving remission. Additionally, DT technology supports weight management, reduces insulin resistance, and improves related metabolic parameters such as blood pressure and lipid profiles (18, 19).

This DT technology represents a significant shift towards precision medicine in T2D care by focusing on individual needs rather than relying on generalized dietary advice. The scalability and integration of DT technology with digital health tools position it as a promising approach for widespread application in T2D management and prevention. In this methods article, we will detail the design and implementation of the DT technology, discuss the underlying ML algorithms, and explore its clinical applications and outcomes. Our findings emphasize the potential of personalized nutrition, driven by advanced digital technology, to transform T2D management, offering a pathway to sustained remission and improved quality of life for patients.

## Materials and equipment

The DT intervention leverages ML algorithms and the Internet of Things (IoT) to create digital replicas, or “twins,” of individuals, enabling personalized management of T2D through predictive modeling (15–17). This system integrates CGM, precision nutrition, physical activity, sleep patterns, and stress management data to deliver tailored recommendations. Implementation requires advanced digital tools, wearable devices, ML algorithms, and laboratory tests.

**Figure 1:**
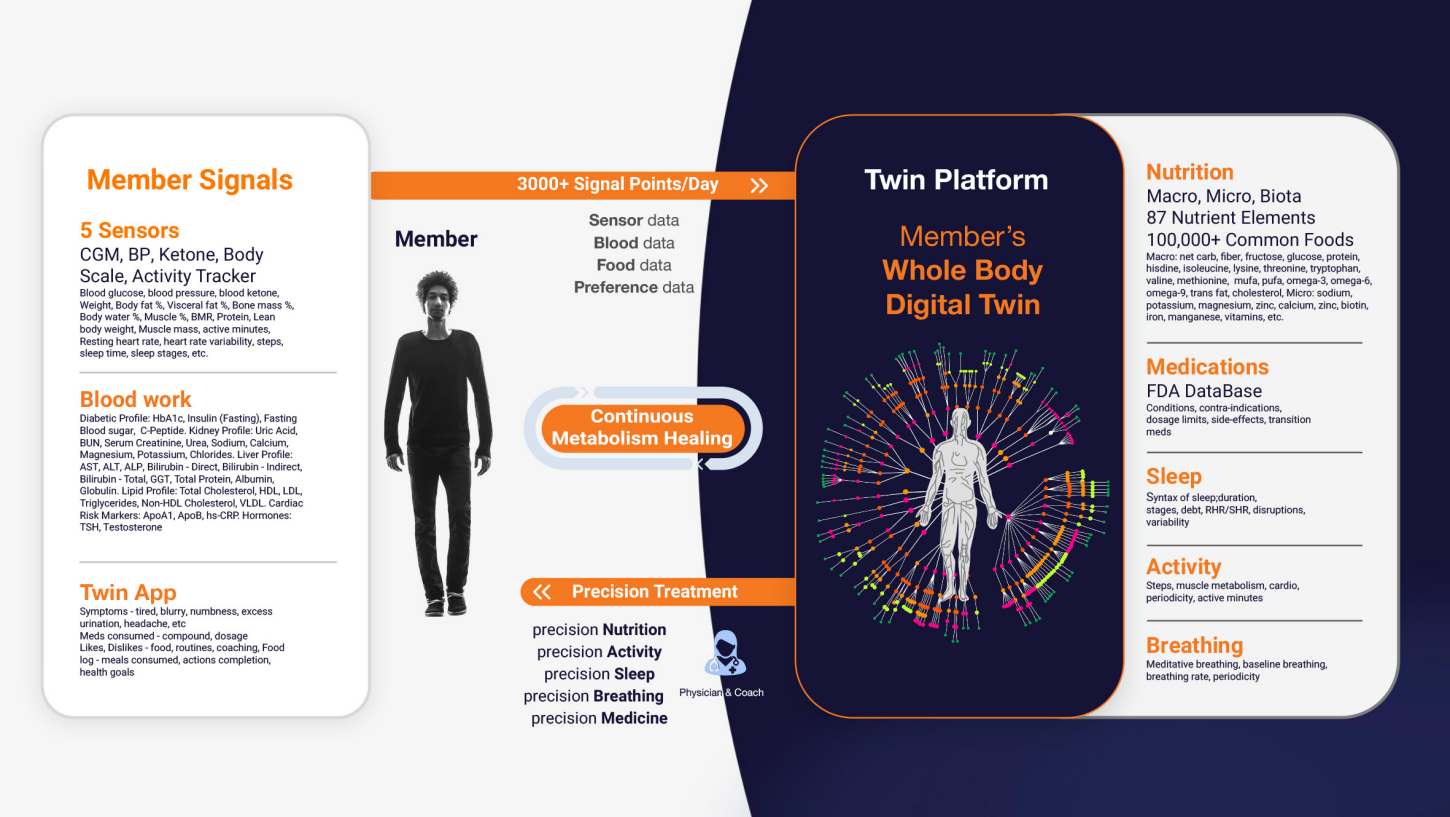
Digital Twin Platform: Member’s Whole Body Digital Twin for Chronic Disease Reversal. This figure illustrates the Digital Twin Platform’s use of real-time sensor data to create a Whole Body Digital Twin, enabling continuous, personalized health interventions across various domains for optimal well-being.

The platform primarily uses the CatBoostRegressor algorithm to predict PPGRs, continuously refining its accuracy with new data. Additionally, Random Forest models enhance predictive capabilities, specifically for PPGR predictions. The process begins with baseline data collection, including demographics, dietary habits, physical activity levels, sleep patterns, and bloodwork. This data is integrated into the DT platform alongside CGM readings to build a personalized metabolic model for each patient, which is used to predict PPGRs. The platform is powered by specialized engines—Nutrition, Medicine, Activity, Sleep, and Breathing—that analyze various health parameters to deliver a holistic approach to T2D management. It also monitors factors related to patient satisfaction, such as taste preferences and engagement (**Figure 1**).

Continuous monitoring via CGM and wearable devices is a key feature of the DT platform. Devices like the Abbott FreeStyle Libre Pro^®^ CGM provide real-time glucose monitoring, while the Fitbit Charge 2^®^ tracks physical activity and sleep patterns. Blood pressure and body composition are also monitored using Bluetooth-enabled devices like the TAIDOC^®^ TD-3140 blood pressure monitor and Powermax^®^ BCA-130 Smart Scale, respectively. These devices sync with the DT platform, allowing seamless health data transmission and analysis. Calibration standards and educational materials are provided to patients to ensure proper use of the monitoring devices and active engagement with the DT technology.

**Figure 2:**
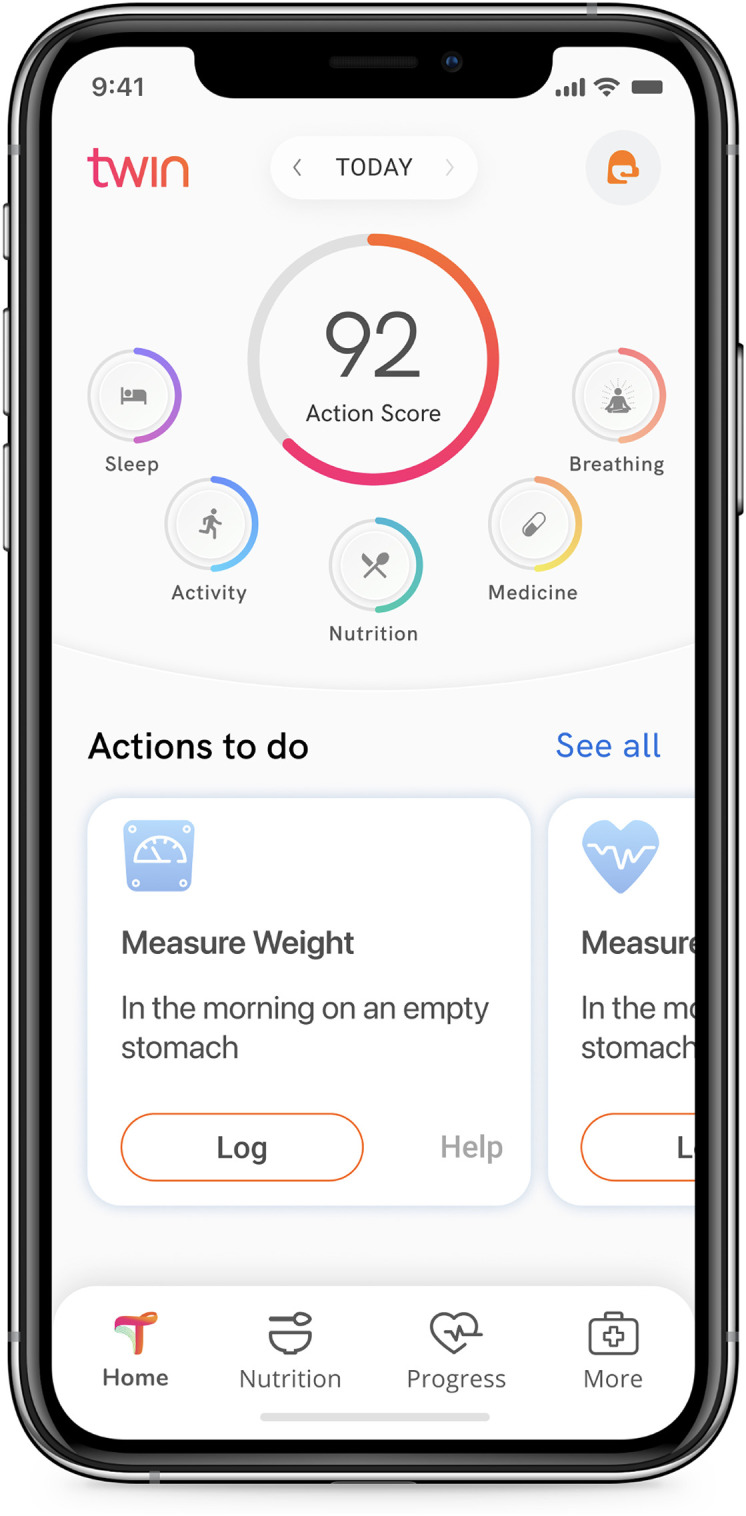
Whole Body Digital Twin^®^ (WBDT) mobile app User Interface. The WBDT Member App interface centers on the daily Action Score, which aggregates data from health modules like Sleep, Breathing, Activity, Nutrition, and Medicine. Users receive actionable steps, such as measuring weight, to stay aligned with their personalized health plan. The app promotes seamless interaction with health metrics, encouraging consistent engagement.

Patients interact with the system through the Whole Body Digital Twin^®^ (WBDT) mobile app (**Figure 2**), available for both Android and iOS, to log meals, receive real-time nutritional recommendations, and monitor their health metrics. The app is supported by a comprehensive food database containing over 100,000 food items from the United States Department of Agriculture’s (USDA) FoodData Central and National Institute of Nutrition (NIN), which assists users in accurately logging dietary intake.

At the outset of the program, participants undergo comprehensive blood tests, including panels for HbA1c, fasting glucose, lipid profiles, and liver function. These tests are repeated periodically to track metabolic changes and guide treatment adjustments.

The DT platform includes a telehealth component for remote patient monitoring and communication with healthcare providers, enabling real-time consultation and timely adjustments to treatment plans. Medications are individualized based on CGM readings and adjusted following standardized protocols. The DT Care app is used by healthcare providers to monitor patient progress, manage medications, and provide ongoing support.

Through continuous monitoring and data-driven adjustments, the DT technology is particularly effective in reducing hyperinsulinemia, improving insulin sensitivity, and supporting T2D remission. It also aids in managing broader metabolic conditions such as obesity, hypertension, and dyslipidemia. Long-term monitoring enables proactive treatment adjustments and early intervention, helping prevent metabolic deterioration.

## Methods

The primary objective of this study was to implement and validate DT technology for personalized management of T2D. The DT system was designed to predict and optimize PPGRs, reduce hyperinsulinemia, enhance insulin sensitivity, and facilitate T2D remission. The validation process evaluated the accuracy of predictive algorithms, the effectiveness of personalized dietary interventions, and clinical outcomes in patients.

### Data collection and setup

The DT program was operationalized through patient onboarding, which included instructions on using monitoring devices and logging dietary intake via a mobile app. Comprehensive data collection involved gathering demographic details, dietary habits, physical activity, sleep patterns, and bloodwork. Each patient was equipped with CGMs, sensor watches, blood pressure monitors, and body composition scales, synchronized with the DT platform. The initial setup took 1–2 hours per patient, providing the foundation for building the DT. Data preprocessing involved cleaning raw CGM and sensor data, removing outliers, correcting errors, and handling missing values through interpolation. Dietary logs were standardized to ensure data consistency, critical for model development and analysis.

In parallel, continuous time series glucose data were collected from CGMs. Metrics such as weight, height, HbA1c, medications like Gliclazide, Glimepiride, and Metformin, and other clinical parameters were recorded during doctor’s visits. Data from activity trackers, such as steps, minutes asleep, and active minutes, were also collected. Nutritional data, including calories, carbohydrates, proteins, fats, fibers, glycemic index, and glycemic load, were calculated from the Precision Nutrition Database, while meal types (e.g., breakfast, lunch) and quantities were logged by patients.

The DT platform addresses variability in data quality through multiple safeguards and adaptive algorithms. It employs real-time error detection to flag anomalies, continuously updates predictions using recent trends, and leverages long-term patterns and cohort-based models to ensure reliable outputs despite intermittent data. A feedback loop prompts users to validate and correct discrepancies, supporting robust, personalized recommendations.

Data reliability is ensured through automated validation, user feedback for corrections, baseline validation to monitor deviations, and reminders for sensor calibration. Cross-validation across biometric sources further enhances data consistency, ensuring accurate predictions.

Outliers are managed through automated detection of deviations from historical trends, data imputation to fill gaps, and weight adjustments to minimize the impact of anomalies. The feedback loop engages users to confirm flagged data, capturing true responses while excluding errors for reliable predictions.

### Ethical and legal compliance in data privacy, security, and patient confidentiality

The DT platform adheres to stringent ethical and legal standards through comprehensive protocols for informed consent, data protection, and compliance with international regulations like Health Insurance Portability and Accountability Act (HIPAA), General Data Protection Regulation (GDPR), and India’s Personal Data Protection Bill (PDPB). Informed consent is obtained digitally, with options for patients to opt in or out of specific data-sharing functionalities, and consent preferences can be modified at any time. The platform ensures secure telehealth sessions through end-to-end encryption, and consent discussions are documented (with permission) to verify compliance. Data is stored locally based on regional regulations (e.g., within the EU for GDPR and in certified US centers for HIPAA). For cross-border transfers, secure mechanisms like Standard Contractual Clauses (SCCs) are used. Data is minimized, anonymized, or pseudonymized where feasible, and access is controlled using Role-Based Access Control (RBAC), with regular audits to monitor compliance.

Sensitive data is encrypted using AES-256 for storage and Transport Layer Security (TLS) 1.2+ for transmission, with multi-factor authentication (MFA) enforced for all users. The platform also includes safeguards against device malfunctions, and patient rights, such as viewing, downloading, or deleting data, are upheld globally. In case of data breaches, affected patients and regulatory authorities are notified within 72 hours, as per GDPR. Ethical oversight is provided by a dedicated Ethics Review Board, and patient feedback is routinely collected to ensure the system aligns with ethical guidelines.

Additionally, in the clinical study, all participants provided written informed consent detailing the study’s nature and wearable device usage. Patient data was anonymized using unique identifiers, with personal data stored separately in encrypted, HIPAA-compliant databases accessible only to authorized personnel. Security measures, including Advanced Encryption Standard - 256 bit (AES-256) encryption and TLS, were employed to protect data at all stages, and regular audits were conducted to ensure compliance.

### Machine learning model

**Figure 3:**
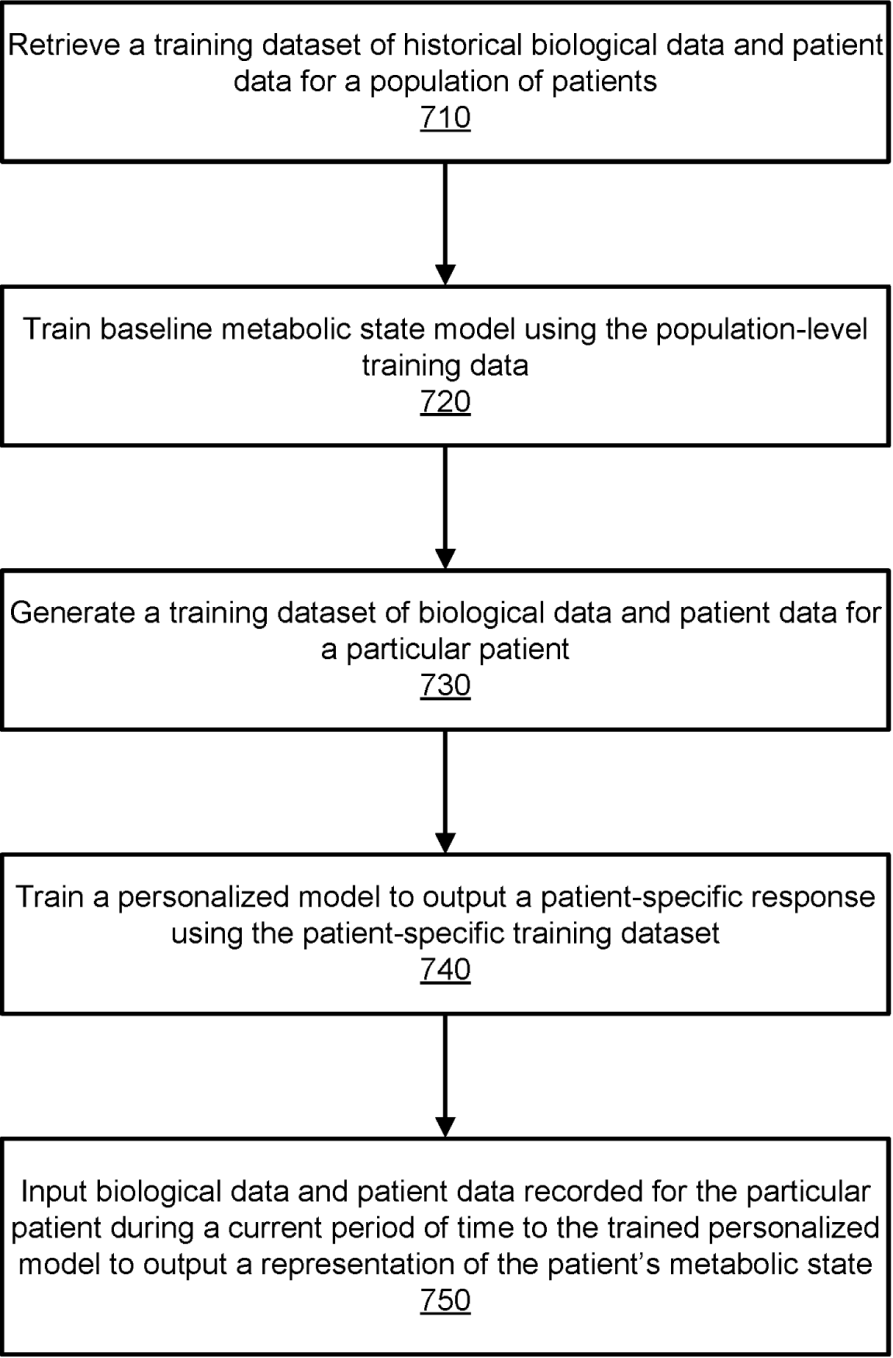
Flowchart illustrating the process for training a machine-learned model to output a representation of a patient’s metabolic health. This figure outlines the process used by the digital twin module to train a machine-learning model for predicting a patient’s metabolic state. The process begins with retrieving historical biological and patient data (710) to train a baseline model (720) reflecting population-level trends. Next, a patient-specific dataset (730) is generated, and a personalized model (740) is trained to predict individual metabolic responses. Finally, current biological and patient data are inputted into the trained model (750) to output real-time metabolic states. Data sources include lab tests, sensor data, and patient-recorded measurements, enabling precise metabolic monitoring and health predictions. This figure is taken from the patent US 2021/0196195 A1.

**Figure 4:**
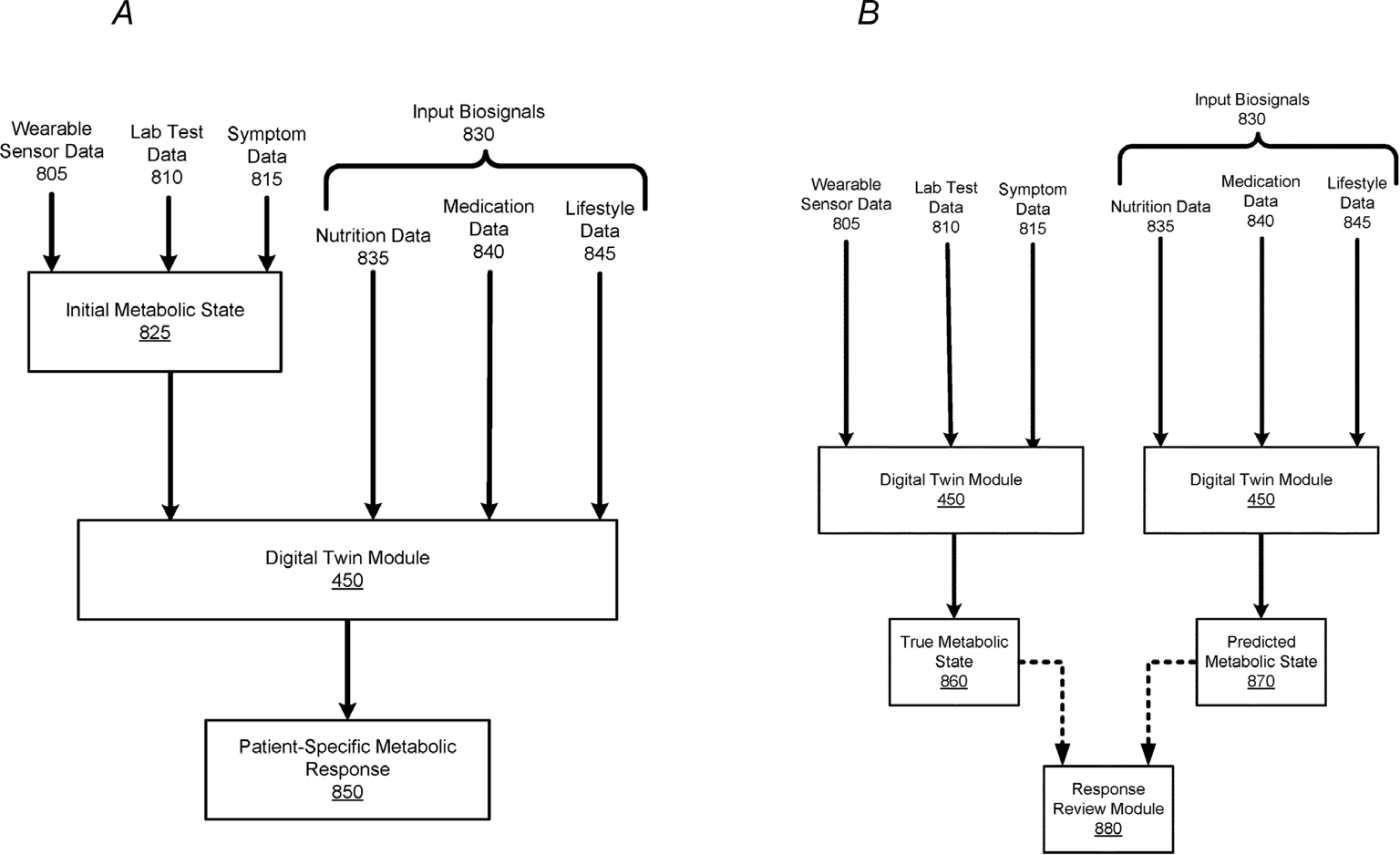
Process of Predicting and Verifying Patient-Specific Metabolic Responses Using a Machine-Learned Model. **(A)** This figure depicts the process of implementing a machine-learned model to predict a patient’s specific metabolic response. The model uses wearable sensor data (805), lab test data (810), and symptom data (815) to establish an initial metabolic state (825). Once sufficient training data for a patient exists, the digital twin module (450) can predict the patient’s metabolic response (850) based on input biosignals (830), such as nutrition (835), medication (840), and lifestyle data (845). The predicted metabolic response reflects changes in the patient’s health, corresponding to the input biosignals. **(B)** This figure illustrates the comparison process between a patient’s predicted metabolic state and true metabolic state. During a given time period, wearable sensor data (805), lab test data (810), and symptom data (815) are used to generate a patient’s true metabolic state (860) via the digital twin module (450). Simultaneously, input biosignals (830), such as nutrition, medication, and lifestyle data, are processed to predict the patient’s metabolic state (870). The Response Review Module (880) compares the true and predicted metabolic states to identify discrepancies, helping detect any errors in the biosignal inputs that might have contributed to the differences. This figure is taken from the patent US 2021/0196195 A1.

**Figure 5:**
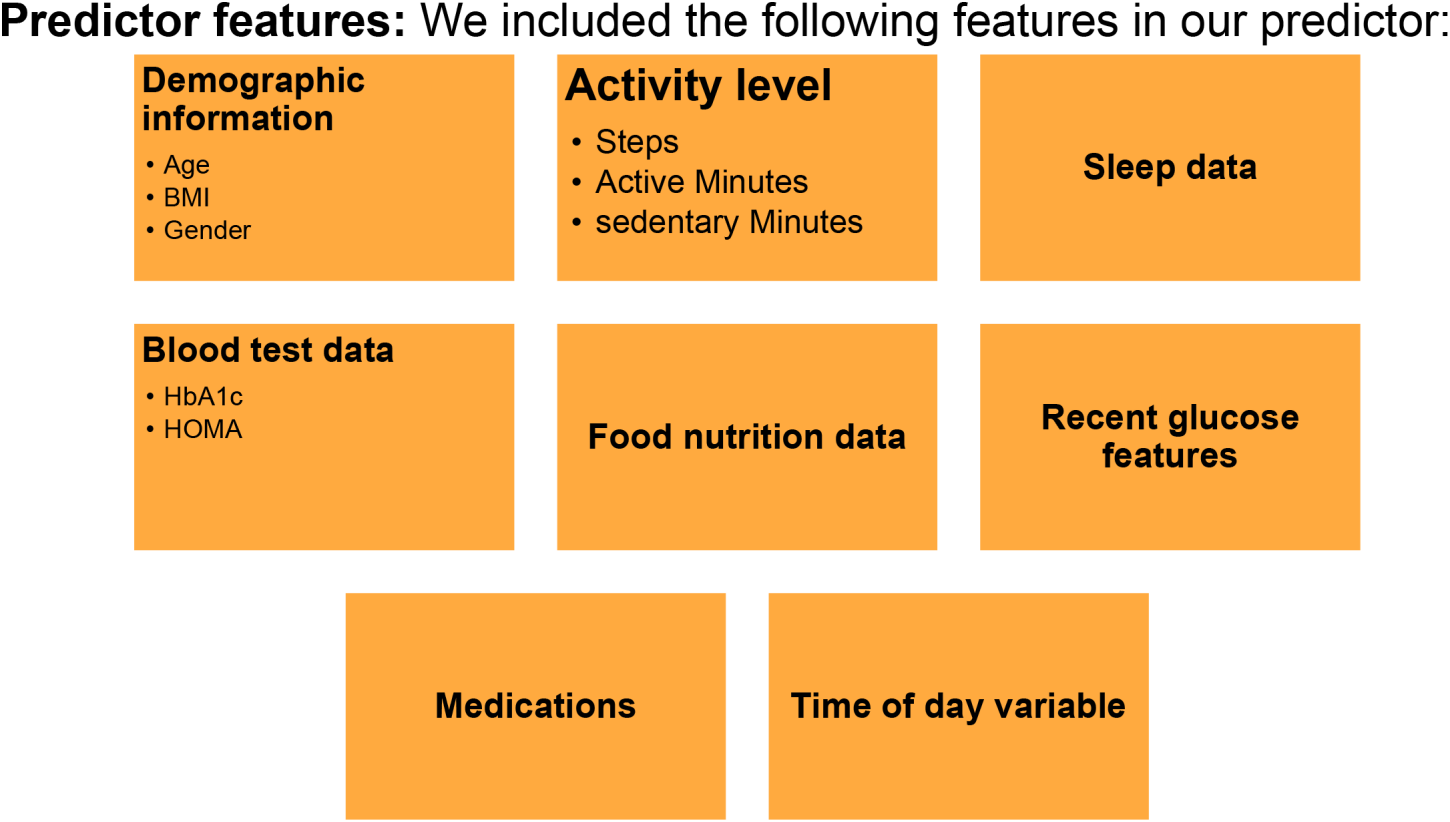
Predictor Features for Digital Twin Model Development. The predictor model includes demographic info (age, sex, BMI, gender), activity level (steps, active/sedentary minutes), sleep data, blood test results (HbA1c, HOMA), food nutrition data, recent glucose features, medications, and time of day.

A personalized PPGR prediction model (**Figures 3**, **4A, B**), the Glucose Impact ML Model, was developed using patient-specific data. This model, leveraging features such as meal composition, historical glycemic responses, physical activity, and sleep patterns, allowed individualized predictions of glucose peaks (‘glucoseMax’) and the relative impact of various foods. Input features (**Figure 5**) included 37 variables, such as calories, carbohydrates, proteins, fats, fiber, glycemic index, recent glucose trends, medications, sleep, and activity metrics. The model predicted glucose peaks within specific meal-time windows and normalized them against each patient’s baseline glucose level, which was penalized based on medication use to reflect artificially reduced glucose baselines.

Two machine learning algorithms, CatBoostRegressor and Random Forest, were employed for prediction tasks. CatBoostRegressor was chosen for its ability to handle categorical variables and reduce overfitting, using gradient boosting to enhance model accuracy. Random Forest was selected for its robustness in modeling non-linear relationships between variables such as time of day, food types, and glucose levels. The key model parameters included the number of trees (n_estimators), typically set between 100 and 300, and the maximum depth (max_depth), which ranged from 3 to 7 to control tree complexity. The minimum number of samples required to split an internal node (min_samples_split) was set to 2, ensuring that nodes only split when at least two samples are present. Additionally, feature subsampling (max_features) was used to define the number of features considered at each split, commonly set to the square root of the total number of features, to prevent overfitting and enhance model generalization. Random Forest Ensemble Equation: Each tree in the Random Forest produces an independent prediction. The final prediction is the average of all individual trees: $\begin{array}{l} \hat{y}=(1/T)\sum h_{t}(x)\\ t=1 \end{array}$

where: ŷ = Final ensemble prediction; T = Total number of trees in the forest; h_t_(x) = Prediction from the t-th decision tree for a given input x.

The models were implemented using Python (v3.8) with relevant libraries, including CatBoost (v0.26), scikit-learn (v0.24.2), and pandas (v1.3.3). Statistical analyses were conducted using R software (v4.3.1), and visualizations were created using Matplotlib (v3.4.3).

### Model assumptions

Several assumptions were made to enhance model performance:

- Independence of Residuals: Residuals between predicted and observed glucose levels were assumed to be independent and normally distributed.
- Feature Stability: Relationships between input features and PPGR outcomes were assumed to remain stable over time.
- Minimal Multicollinearity: The models were structured to minimize multicollinearity and ensure independent contributions from each feature.

### Model performance metrics

Model performance was evaluated using key metrics (**Table 1**) such as mean squared error (MSE), root mean squared error (RMSE), mean absolute error (MAE), R-squared (R²), and the area under the receiver operating characteristic curve (AUC-ROC) for classification tasks. MSE, defined as MSE = (1/N) * Σ (y_i − ŷ_i)², was used to quantify average squared differences between actual and predicted values, where lower values indicate better predictive accuracy. For glucose predictions, an MSE range of 50 to 100 is generally acceptable for meal-induced glucose spikes, and our model achieved an MSE between 45 and 55, demonstrating strong predictive performance. Similarly, RMSE, calculated as √(1/N Σ(yi - ŷi)²), provides an overall measure of prediction quality and is sensitive to large errors. An RMSE of 24.96 mg/dL for glucose peak predictions indicates that the predicted glucose levels were, on average, within 24.96 mg/dL of the actual values.

**Table 1:** Model performance metrics.

Metric	Value	Clinical Relevance
RMSE	24.96 mg/dL	Shows that predicted glucose peaks are within 24.96 mg/dL of actual values. Suitable for evaluating glucose variability. Supports accurate decision-making around medication and dietary adjustments. Suggests that the model is capable of accurately predicting glucose excursions, thus preventing adverse glycemic events such as hyperglycemia or hypoglycemia.
MAE	17.21 mg/dL	Average absolute deviation between predicted and actual values, indicating precision of dietary recommendations. An MAE below 20 mg/dL is considered clinically acceptable for guiding dietary and lifestyle recommendations. Supports decision-making about meal planning and carbohydrate intake. This level of precision also supports maintaining glucose within the recommended time-in-range (TIR) of 70–180 mg/dL, thereby improving glycemic control.
R2	> 0.85	High R2 indicates strong predictive power. This means that the model can reliably predict the impact of various factors (such as meal composition and timing) on blood glucose levels. For clinicians and patients, a high R² value translates to more accurate predictions of postprandial glucose responses, enabling more precise dietary and medication recommendations. This level of predictive power is crucial in preventing unexpected glycemic excursions and achieving optimal glycemic control.
AUC-ROC	> 0.85	Measures the model’s ability to classify glucose spikes accurately, with a high true positive rate and a low false positive rate. An AUC-ROC above 0.85 supports identifying potential hyperglycemic events and enabling timely interventions such as adjusting insulin dosages or modifying meal recommendations to prevent hyperglycemic episodes.

This table summarizes the key performance metrics used to evaluate the personalized postprandial glycemic responses (PPGR) prediction model. Root Mean Squared Error (RMSE) and Mean Absolute Error (MAE) quantify the model’s prediction accuracy, with lower values indicating better precision. R-squared (R²) measures the proportion of variance explained by the model, where values above 0.85 indicate strong predictive power. The Area Under the Receiver Operating Characteristic Curve (AUC-ROC) assesses classification performance, with an AUC-ROC score of >0.85 demonstrating reliable identification of glucose spikes.

MAE, defined as (1/N) Σ|yi - ŷi|, measures the average magnitude of errors without considering their direction and is less sensitive to large deviations. An MAE of 17.21 mg/dL suggests that, on average, the predicted glucose levels deviated by ±17.21 mg/dL from actual values, with 91% of predictions falling within 40 points of actual values. R-squared (R²), given by 1 - Σ(yi - ŷi)²/Σ(yi - )², quantifies the proportion of variance in the dependent variable explained by the model, with values closer to 1 indicating better predictive power. Our model achieved an R² value of >0.85, indicating that it captured over 85% of the variability in glucose levels, demonstrating strong predictive power.

For classification tasks, AUC-ROC was employed to assess the model’s ability to differentiate between positive and negative classes, with scores ranging from 0.5 (no discrimination) to 1 (perfect discrimination). Our model achieved an AUC-ROC score of >0.85, indicating excellent discriminatory capability in distinguishing between glucose spikes and non-spikes. To confirm statistical significance, exact p-values (p < 0.05) were calculated, along with 95% confidence intervals and effect sizes to quantify the strength of the observed relationships.

We used the Multiple Imputation by Chained Equations (MICE) method to handle missing data, which involves generating five imputed datasets based on observed patterns. Each variable with missing values was modeled using linear regression (for continuous variables) or multinomial logistic regression (for categorical variables). Results from the imputed datasets were pooled using Rubin’s Rules to account for uncertainty and variance.

To assess the robustness of the findings, sensitivity analyses were conducted, including complete case analysis, testing with different imputation methods (Predictive Mean Matching, Random Forest), substituting extreme values, and evaluating the impact on key outcomes like HbA1c and glycemic variability. These analyses showed that the study’s conclusions remained consistent, confirming that the imputation process did not significantly alter the results.

### Cross-validation and overfitting prevention

To ensure model robustness and minimize overfitting, k-fold cross-validation (k=5) was implemented with a two-week gap between training and validation data to simulate real-world fluctuations. In this setup, the data was divided into five folds, with each fold serving as the validation set once while the remaining four were used for training. The results were averaged across the folds to enhance generalization and avoid overfitting.

Additionally, an 80-20 split was used initially, with 80% of the data allocated for training and 20% for validation. To optimize model performance, grid search was employed to fine-tune hyperparameters such as learning rate and tree depth. Regularization techniques, including L2 regularization and early stopping, were applied to mitigate overfitting. The model was continuously retrained with new data to maintain real-time accuracy, reducing variance and improving long-term performance.

### Hyperparameter selection process

1. Optimization Strategy:
  ○ Hyperparameter tuning was conducted using grid search and randomized search approaches.
  ○ For the 1-Day Glucose (1DG) model, the primary hyperparameters optimized include:
    ▪ Number of Trees (n_estimators): Typically ranged between 100 and 500.
    ▪ Learning Rate (learning_rate): Set between 0.01 and 0.1.
    ▪ Max Depth (max_depth): Set between 3 and 5.
    ▪ Subsample: Set to 0.8 to reduce overfitting.
2. Nested Cross-Validation:
  ○ Nested cross-validation involved two levels:
    ▪ Inner Loop: Used for hyperparameter tuning.
    ▪ Outer Loop: Used for performance evaluation.
  ○ This strategy ensured that hyperparameters were optimized on separate folds, minimizing data leakage and ensuring reliable performance evaluation.

### Model-specific hyperparameters

1. CatBoostRegressor and Random Forest were used for predicting glucose peaks and variability:
  ○ CatBoostRegressor: Effective in handling categorical variables and reducing overfitting using gradient boosting.
  ○ Random Forest: Utilized for its robustness in modeling non-linear relationships between variables such as food type, meal timing, and glucose trends.

### Generating and validating dietary recommendations

1. Overview of the Dietary Recommendation System (DT System)

○ The DT system uses machine learning to predict personalized PPGRs for various foods and meal configurations, generating real-time dietary recommendations to optimize glycemic control and support T2D remission. By integrating patient data, CGM, and predictive modeling, the DT system adapts dynamically to individual metabolic responses and preferences, adjusting recommendations based on real-time feedback and lifestyle patterns.

2. Data Input and Feature Engineering

The dietary recommendation system requires comprehensive input data to build accurate and personalized predictions. The primary inputs include:

Patient Profile:
  ○ Baseline Clinical Parameters: Age, gender, BMI, waist circumference, insulin sensitivity index, and duration of diabetes.
  ○ Metabolic Markers: Fasting glucose, HbA1c, lipid profile, and blood pressure.
  ○ Medication Usage: Type and dosage of glucose-lowering medications (e.g., Metformin, GLP-1 agonists, SGLT-2 inhibitors).
  ○ Exercise and Sleep Patterns: Physical activity frequency, step counts, sleep duration, and quality.
Meal and Nutrition Data:
  ○ Macronutrient Composition: Proportions of carbohydrates, proteins, and fats.
  ○ Glycemic Index (GI) and Glycemic Load (GL): Used to estimate the glycemic impact of each food item.
  ○ Meal Timing and Portion Size: Timing, portion size, and food combinations (e.g., breakfast, lunch, dinner, or snacks).
Real-Time Data:
  ○ Continuous Glucose Monitoring (CGM): Real-time glucose fluctuations before and after meals.
  ○ Activity Levels: Steps, active minutes, and sedentary periods.
  ○ Sleep Patterns: Sleep efficiency, disturbances, and patterns.
Feature Engineering Process:
  ○ The input data is transformed into a high-dimensional feature set that captures interactions between food items, their timing, and patient-specific responses. For example:
    ▪ Carbohydrate-to-Protein Ratio: To measure the impact of macronutrient combinations on glycemic variability.
    ▪ Time Since Last Meal: Represents how meal spacing affects subsequent PPGR.
    ▪ Previous Glucose Trends: Captures the rate of change in glucose levels over time to reflect temporal dependencies.

3. Method for Generating Dietary Recommendations

The core of the DT system is a machine learning model that predicts the expected PPGR for different food items and combinations. This model is built using a hybrid architecture that integrates two major components:

Gradient Boosting Machine (GBM):
  ○ Purpose: Handles structured and static data like nutrient composition, patient demographics, and baseline metabolic parameters.
  ○ Key Parameters:
    ▪ Number of Trees: Typically set between 100 to 500.
    ▪ Learning Rate: Set between 0.01 to 0.1 to control the contribution of each additional tree.
    ▪ Max Depth: Set to 3-5 to manage model complexity.
Long Short-Term Memory (LSTM) Network:
  ○ Purpose: Captures sequential dependencies and temporal relationships between consecutive glucose readings, meals, and physical activity.
  ○ Key Parameters:
    ▪ Number of Units: Set between 50 to 200 depending on the model’s complexity.
    ▪ Dropout Rate: Set between 0.2 and 0.5 to prevent overfitting.
    ▪ Learning Rate: Fixed at 0.001 for Adam optimizer.
  ○ Data Integration: Inputs include nutritional data (e.g., macronutrients, glycemic index), patient features (e.g., age, BMI, medication), meal timing, and real-time CGM data. Additional features like “carbohydrate-to-protein ratio” and “previous glycemic trend” are created through feature engineering.
  ○ PPGR Prediction Model: Combines Gradient Boosting Machine (GBM) and Long Short-Term Memory (LSTM) networks. The GBM handles static features like nutritional content, while the LSTM captures temporal dependencies. The model equation is defined as: PPGR(t) = f(Σ wi · ϕ(ht, xi))
      where:
    - PPGR(t) = Predicted postprandial glucose response at time t.
    - wi = Learned weight for each feature.
    - ϕ(ht, xi) = Feature interaction function that considers temporal dependencies (ht) and static features (xi).
    - f = Predictive function that maps the combined features to the PPGR value.

4. Dietary Recommendation Engine

The dietary recommendation engine processes the PPGR predictions to generate meal recommendations that align with the patient’s glycemic targets and nutritional requirements. It follows a multi-objective optimization strategy that incorporates the following steps:

Optimization Function: The recommendation process is guided by an optimization function that seeks to minimize glycemic variability while maximizing nutritional quality and adhering to patient preferences. The objective function is defined as:
Recommendation=arg min_food(PPGR_predicted+λ1·Nutrient Variability+λ2·Patient Preferences)
  where:
  argminfood means the system is searching for the food item that results in the smallest value of the objective function
  PPGR_predicted = Predicted postprandial glycemic response.
  λ1 = = Regularization parameter for nutrient variability.
  λ2 = Regularization parameter for patient-specific preferences.
Personalized Recommendation Criteria:
  ○ Minimization of Predicted PPGR: Foods and meal combinations with lower predicted PPGRs are prioritized.
  ○ Nutrient Density: Meals must maintain a balance of proteins, vitamins, and essential nutrients to support overall health.
  ○ Patient Preferences and Restrictions: Incorporates dietary restrictions (e.g., vegetarian diets, food allergies) and lifestyle choices.
Real-Time Adjustment Mechanism:
  ○ The DT system integrates real-time CGM feedback to iteratively refine the recommendations. If a meal generates a higher-than-expected glycemic excursion, the model is retrained using the new data to update future predictions and recommendations.
  ○ A Corrective Model is used to adjust for unexpected variations, incorporating recent meal responses and glycemic outcomes.

5. Ranking and Selection: The Glucose Impact Model enabled classification of foods into three categories:

- Green For You (GFY): Foods with minimal glycemic impact, recommended for unrestricted consumption.
- Orange For You (OFY): Foods with moderate impact, to be consumed sparingly.
- Red For You (RFY): Foods with high impact, recommended to be avoided.
  These recommendations were derived from a Nutrition Rules Table (NRT) and dynamically adjusted based on each patient’s evolving metabolic state.

6. Validation and Evaluation of Dietary Recommendations

 The validation process includes short-term and long-term evaluations to ensure that the recommendations produce desired clinical outcomes:
  ○ Short-Term Validation: Recommendations are validated using real-world CGM data. Metrics include:
    ▪ MAE: Average error between predicted and actual PPGR.
    ▪ RMSE: Precision of glucose peak predictions.
    ▪ Time-in-Range (TIR): Time within the target glucose range (70-180 mg/dL).
  ○ Long-Term Validation:
    ▪ Glycemic Variability: Standard deviation of daily glucose readings.
    ▪ Reduction in HbA1c: Monitored over 3-month intervals.
    ▪ Patient Satisfaction: Surveys assess adherence and ease of use.
  ○ Patient-Centric Outcomes:
    ▪ The DT system evaluates patient engagement and ease of implementation through feedback channels integrated into the platform.
    ▪ Adjustments to the dietary recommendations are made based on feedback and real-time data, ensuring that the recommendations are both clinically effective and practical for patients.

7. Example of Recommendation Generation

 ○ For a patient with high PPGR to carbohydrate-rich meals:
   ▪ Initial Recommendation: Substitute high-PPGR foods (e.g., white rice) with low-PPGR alternatives (e.g., quinoa) and increase protein and fiber.
   ▪ Real-Time Adjustment: If spikes persist, adjust portion sizes or recommend additional changes, such as including healthy fats.

8. Validation of Effectiveness

 ○ Recommendations are validated using:
   ▪ Reduction in Hyperglycemic Episodes: Time spent above 180 mg/dL.
   ▪ Improvement in Metabolic Markers: Lower HbA1c and improved insulin sensitivity.
   ▪ Patient-Centric Outcomes: Increased satisfaction, adherence, and quality of life.

### Continuous monitoring and model maintenance

Model predictions were regularly evaluated against actual CGM data to maintain precision. Calibration and retraining were conducted periodically to account for sensor inaccuracies or physiological changes. Proprietary technologies, outlined in key patents (e.g., US11185283B2, US11957484B2) (16, 17), were updated as advancements in machine learning and digital twin technology occurred.

The DT platform provides a scalable, data-driven solution for personalized T2D management by leveraging advanced digital tools and machine learning models. It enables precise, real-time dietary and lifestyle recommendations, offering a significant improvement in glycemic control and the potential for sustained T2D remission.

## Results

Below is a summary of our ongoing open-label RCT titled “Randomized Controlled Trial of Twin Precision Treatment (TPT): A Novel Digital Twin-Based Precision Approach for Reversing Diabetes”. The results are based on initial published data from the trial (18), which are cited to support the methodology.

### Study design

This study adhered to the Declaration of Helsinki, approved by the Medisys Ethics Review Board (MCERB/2020/07), and was registered with the Clinical Trials Registry-India (CTRI/2020/08/027072). It was designed as a two-year study with a three-year extension, making it a five-year trial to assess the outcomes of DT technology in managing T2D.

### Trial participants

Eligible participants were aged 18–70, diagnosed with T2D for ≤8 years, with normal liver and kidney function, and proficient in smartphone use. Exclusion criteria included pregnancy, non-T2D diabetes, prior or planned bariatric surgery, and significant psychiatric disorders. Participants were recruited from four diabetes centers in India, and all provided informed consent.

### Randomization and blinding

Participants were randomized using a computer-generated sequence in a 3:1 ratio to the DT group (n=250) or the standard care (SC) group (n=86). Seventeen participants in the DT group withdrew before the intervention, leaving 319 participants: 233 DT and 86 SC participants for intent-to-treat (ITT) analysis.

### Outcomes

Primary outcomes included changes in HbA1c and T2D medication dosages from baseline to two years, measured at 90-day intervals. Secondary outcomes included T2D remission rates (remission was defined as an HbA1c <6.5% without glucose-lowering medications for at least 3 months) (20).

### Intervention Group (DT)

The DT group received personalized dietary recommendations based on AI-driven predictions of glucose responses. The intervention, supported by health coaches, aimed to modify behavior through AI guidance. The DT program consisted of three phases: a 90-day “Restrictive Phase” that limited high PPGR foods, followed by a 90-day “Reintroduction Phase,” and a long-term “Maintenance Phase” with ongoing AI-guided dietary adjustments. Most medications, except metformin and sitagliptin, were discontinued and titrated as needed.

### Standard care group

SC participants received conventional T2D management, including quarterly clinical assessments and lab tests. Treatment focused on medication, lifestyle modifications, and consultations with physicians and nutritionists. Lifestyle advice emphasized a balanced diet, physical activity (150 minutes per week), and behavioral support, including goal-setting and blood glucose monitoring.

### Statistical analysis

The sample size was calculated to detect a ≥1% difference in HbA1c between the DT and SC groups, assuming an SD of 1.83, 90% power, and a significance level of 0.05. Accounting for a 20% dropout rate, the study included 250 DT and 86 SC participants. Seventeen participants in the DT group withdrew before the intervention, leaving 319 participants: 233 DT and 86 SC participants for ITT analysis. The remission sample size was smaller than that for HbA1c reduction. Data analysis followed the ITT principle, with missing data imputed using the mean and verified via multiple imputation. Continuous variables were tested for normality by Shapiro-Wilk test and presented as mean (SD), while categorical variables were shown as counts and percentages. Baseline characteristics were compared using independent t-tests and Chi-square tests. Post-intervention outcomes at two years were analyzed using t-tests, and paired t-tests were used for within-group comparisons. A type 1 error rate of 0.05 was applied, with the false discovery rate controlled by the Benjamini-Hochberg procedure. Sensitivity analyses included adjustments for baseline covariates through multivariable models and propensity scores. Statistical analyses were performed using SPSS version 28.0, with p-values <0.05 considered significant.

Below is a summary of our ongoing open-label RCT results at the one-year mark (18). The initial sample included 319 patients: 233 in the DT group and 86 in the SC group. At the 1-year follow-up, 37 patients (15.9%) dropped out from the DT group (leaving 196), and 16 patients (18.6%) dropped out from the SC group (leaving 70). The dropout rates were similar in both groups, indicating it was not related to the DT intervention. DT-enabled personalized nutrition significantly lowers A1C levels in patients with T2D. In the DT group, the mean HbA1c improved remarkably from 9.0 (± 1.9) to 6.1 (± 0.7) over one year (P<0.001), whereas the standard care (SC) group using traditional dietary interventions showed a non-significant change from 8.5 (± 1.9) to 8.2 (± 1.6) (P=0.051) (18). After one year, 94% of the DT group discontinued all T2D medications, with 72.7% achieving T2D remission, compared to none in the SC group (18). The DT technology has also demonstrated significant success in improving Metabolic Dysfunction-Associated Fatty Liver Disease (MAFLD). The proportion of patients with normal Nonalcoholic fatty liver disease-Liver Fat Score (NAFLD-LFS) increased from 11.8% to 67.4% in the DT group, whereas it decreased from 16% to 9.9% in the SC group over one year (P<.00001) (18). Similarly, the proportion of patients with normal fatty liver index (FLI) increased from 13.6% to 54.3% in the DT group compared to a reduction from 15.9% to 13.4% in the SC group (P=0.0003) (18). At one year, the proportion of patients with normal Framingham Steatosis Index (FSI) increased from 13.1% to 61.1% in the DT group while remaining stable in the SC group (P=0.0008) (18). Liver fibrosis scores also improved, with the proportion of patients with normal Nonalcoholic fatty liver disease Fibrosis Score (NFS) increasing from 77.6% to 94.4% in the DT group compared to an increase from 60.7% to 65.5% in the SC group (P=0.58) (18). The DT group also showed significant reductions in liver fat percentage by Magnetic Resonance Imaging Proton Density Fat Fraction (MRI-PDFF) (5.5 ± 4.7% *vs*. 10.9 ± 6.8%, P<.001), with more patients achieving a liver fat content <6% than in the SC group (83.2% *vs*. 34%) (18).

DT technology has shown significant cardiovascular benefits as well. There was a notable reduction in systolic blood pressure (SBP) from 140.2 ± 15.8 to 127.1 ± 8.3 mmHg (P=0.02) and diastolic blood pressure (DBP) from 94.7 ± 11.7 to 86.8 ± 6.4 mmHg (P=0.014) in the DT group over six months (19). Additionally, twelve patients discontinued medications needed to manage hypertension, achieving a remission rate of 60% (19). The DT group also demonstrated a marked shift towards a lower ASCVD risk profile, with 76.6% of participants categorized as low risk at one year compared to 49.1% at baseline. High-risk category reductions were more pronounced in the DT group, decreasing from 8.1% to 0.9% (21).

The DT group demonstrated significant improvements in albuminuria, with 92% of participants exhibiting normal albuminuria at one year, up from 79% at baseline. Macroalbuminuria decreased from 4% to 1% in the DT group, and mean albuminuria levels significantly decreased from 36.5 mg/g to 17 mg/g (P=0.0166) in the DT group while increasing in the SC group (22). Significant improvements were also observed in diabetic neuropathy, with 96.4% of DT patients showing improved touch sensitivity and 63.0% showing better vibration perception. However, the correlation between remission and sensory function recovery was weak, suggesting the need for comprehensive management approaches (23). Among patients initially diagnosed with diabetic retinopathy (DR), 43.75% showed regression with no evidence of DR after the intervention, highlighting the potential for positive retinopathy outcomes through DT technology, though the correlation with T2D remission was minimal (24).

At one year, 73.8% of patients in the DT group achieved a weight loss of ≥5%, and 41.6% achieved a weight loss of ≥10%. Significant differences were observed in mean weight reduction (7.4 ± 5.9 kg *vs*. 0.4 ± 3.6 kg P<0.001), BMI reduction (2.7 ± 2.3 kg/m² *vs*. 0.1 ± 1.8 kg/m² P<0.001), and waist circumference reduction (9.5 ± 7.7 cm *vs*. 1.2 ± 10.8 cm P<0.001) compared to the SC group (18).

The results mentioned above are the one year outcomes of our ongoing RCT which is planned for five years duration. At 18 months, 64.3% of the DT participants who achieved remission at 12 months maintained their remission status, demonstrating a considerable level of sustainability. The HbA1c levels in the DT group continued to show significant improvement from baseline to 18 months (8.9 ± 1.9 to 6.4 ± 0.9), while the SC group showed only minimal changes (8.5 ± 1.8 to 8.3 ± 1.6). These results highlight the potential for long-term glycemic control in the DT group (25). We will continue to report the outcomes of all the remaining five years to evaluate the long-term sustainability.

We have implemented several strategies to assess long-term remission and prevent relapse. Follow-up periods now extend beyond 18 months to capture trends in glycemic control and intervention durability. Structured relapse prevention protocols, including booster sessions for behavioral reinforcement, dietary adjustments, and continuous patient engagement via digital platforms, are integrated. The DT program uses advanced tools like CGM and predictive analytics for early detection and timely intervention. Behavioral support modules focusing on stress management, motivation, and lifestyle adherence are incorporated to address psychosocial factors. Additionally, comparative analyses with other interventions identify predictors of sustained remission, helping customize protocols for optimal long-term outcomes.

We conducted a real-world study to evaluate the effectiveness of the DT technology, across Indian and American cohorts with T2D (26). Over 90 days, both cohorts exhibited significant improvements in key glycemic and metabolic parameters, including HbA1c, HOMA-IR, TyG index, and Framingham Risk Score (FRS), with outcomes comparable between the two groups. Notably, DT technology proved effective in reducing cardiovascular risk and improving non-glycemic factors such as body weight and systolic blood pressure, demonstrating its efficacy regardless of differing phenotypes. The findings indicate that the DT model provides consistent benefits across diverse populations, demonstrating its adaptability and effectiveness across varied clinical and sociodemographic profiles (26).

Additionally, our 1-year follow-up data in the U.S Real world study further validated the long-term efficacy of the DT intervention. At 1 year, HbA1c decreased significantly from 7.73 ± 1.38 to 6.22 ± 0.62, with 71.9% of participants achieving HbA1c <6.5%. Moreover, T2D reversal, defined as HbA1c <6.5% with no diabetes medications except metformin, was achieved in 55.5% of participants (27).

We are close to reporting the one-year results of an RCT comparing DT-augmented T2D management to usual care in achieving HbA1C < 6.5% in T2D patients, either with metformin monotherapy or without any T2D medications, while also facilitating weight loss and enhancing quality of life and treatment satisfaction. This single-center, open-label randomized controlled trial was conducted at Cleveland Clinic’s Family Health Center in Twinsburg, Ohio, and is registered on ClinicalTrials.gov (NCT05181449).

A case study demonstrated significant improvement in a patient with Polycystic ovary syndrome (PCOS) following a 360-day intervention program with DT technology, showing substantial changes in ovarian morphology and improvement in metabolic milieu and radiological parameters (28).

The expected outcomes demonstrate the potential for significant improvements in glycemic control, weight management, cardiovascular health, and overall well-being, ultimately leading to T2D remission in a substantial proportion of patients.

## Discussion

DT technology represents an innovative and highly personalized approach to the management and potential remission of T2D. By leveraging advanced technologies such as AI, ML, and continuous real-time monitoring, DT technology addresses the limitations of traditional T2D management strategies that rely on generalized dietary recommendations. DT technology has demonstrated significant improvements across key clinical outcomes, emphasizing the effectiveness of personalized interventions in managing T2D. The DT group achieved a significant 2.9% reduction in HbA1c levels compared to a non-significant change in the SC group over one year. After one year, 94% of the DT group discontinued T2D medications, with 72.7% achieving remission. This highlights the superior glycemic control provided by the DT technology (18). DT technology also led to notable improvements in MAFLD, with the proportion of patients with normal NAFLD-LFS increasing from 11.8% to 67.4% in the DT group while decreasing in the SC group. Additionally, there were significant improvements in FLI and liver fat percentage, further demonstrating the DT’s positive impact on liver health (18).Cardiovascular benefits were another key outcome, with significant reductions in systolic and diastolic blood pressure, substantial weight loss, and a shift towards a lower ASCVD risk profile in the DT group (21). Hypertension remission was particularly notable, with 60% of participants able to discontinue their medications (19). The DT technology also effectively managed T2D complications, showing significant regression in microalbuminuria (22), diabetic neuropathy (23) and diabetic retinopathy (24). Weight loss was another significant achievement, with 73.8% of patients in the DT group achieving at least 5% weight loss, and 41.6% losing 10% or more. There were also notable reductions in BMI and waist circumference, far exceeding those seen in the SC group (18). Lastly, the DT technology showed promise in managing PCOS. A case study demonstrated significant improvements in ovarian morphology and metabolic parameters after a 360-day intervention with the DT, suggesting the technology’s broader applicability beyond T2D (28).

Overall, the DT intervention delivers comprehensive improvements in glycemic control, liver and cardiovascular health, weight management, and even conditions like PCOS, establishing it as a highly effective approach for managing T2D and its associated complications.

This study’s findings are contextualized by recent research on PPGRs (11–14). Studies like the PREDICT 1 have demonstrated significant variability in PPGRs among individuals, even among those consuming the same standardized meal (13). Such variability is largely driven by modifiable factors, such as gut microbiome composition, meal timing, and physical activity, rather than genetic factors alone. The DT technology leverages these insights by utilizing advanced ML models, like CatBoostRegressor and Random Forest, to predict PPGRs based on individual data inputs. This aligns with findings from PREDICT 1, which underscore the importance of personalized approaches to dietary interventions and challenge the efficacy of one-size-fits-all dietary recommendations (13). A data-driven approach to personalized nutrition involves metabolic phenotyping to create predictive models. One such model, utilizing ML, predicts PPGRs based on dietary, anthropometric, physical activity, and gut microbiota data (11). Its accuracy has been validated in an independent cohort, demonstrating potential for tailored dietary interventions. While this method effectively lowered glucose levels with personalized diets, it did not link these results to broader health outcomes. Additionally, it focused on non-diabetic and prediabetic populations, not fully addressing the complexities of PPGR prediction in diabetics, who experience greater glycemic variability and different medication use (11). A recent study (12) indicates substantial variability in PPGRs among individuals consuming identical standardized meals. Although 95.1% of participants exhibited higher PPGRs with increased carbohydrate content, the degree of carbohydrate sensitivity varied greatly, highlighting diverse glycemic responses that were independent of carbohydrate intake levels. This study has several limitations, including lower correlation of intraindividual PPGRs compared to the Israeli cohort (11), potentially due to the choice of a standardized meal (bagel and cream cheese) (13). The model’s reliance on complex features such as CGM measurements and fecal analyses, though increasingly accessible, may limit its broader application. Among European adults, internet-delivered personalized nutrition advice led to more significant and appropriate dietary changes than conventional methods. However, the online nature of this study limited the range of measures, with some key health biomarkers, like blood pressure, not recorded. Data were self-reported or collected remotely, introducing potential measurement errors (14).

The impressive results achieved with the DT technology have far-reaching implications for the future of T2D management and precision medicine. By utilizing real-time data and advanced predictive models, this personalized approach offers a substantial advancement in addressing not only glycemic control but also other critical aspects of metabolic health. The DT technology’s success in managing T2D (18), MAFLD (18), hypertension (19), and complications like diabetic neuropathy (23) and retinopathy (24) is particularly noteworthy. One of the most striking implications is the DT technology’s ability to significantly lower HbA1c levels and achieve high remission rates in T2D patients (18). This suggests that personalized nutrition, guided by sophisticated digital tools, can fundamentally change the course of T2D, offering a viable alternative to traditional management strategies that often fall short of achieving long-term remission and leave patients dependent on pharmacotherapy.

Additionally, the DT technology’s broader benefits in cardiovascular health (21), liver function (18), and weight management (18) indicate its potential as a comprehensive solution for managing the complex challenges of metabolic syndrome. The inclusion of a case demonstrating significant improvement in a patient with PCOS further highlights the versatility and effectiveness of this approach (28). The DT technology’s ability to improve ovarian morphology and metabolic parameters in PCOS suggests that it could also play a vital role in managing other endocrine and metabolic disorders. The high remission rates observed in this study suggest that personalized nutrition, when guided by advanced digital tools like the DT, can potentially reverse the course of T2D in many patients. This is particularly important given the limitations of traditional management strategies, which often fail to achieve long-term remission and leave patients reliant on pharmacotherapy.

The DT technology offers a unique and comprehensive approach to diabetes management, setting itself apart from other digital tools such as Livongo (29), Omada Health (30), One Drop (31), and mySugr (32). Unlike these platforms, the DT system constructs a highly detailed, patient-specific virtual model that continuously monitors and predicts metabolic responses using real-time biosignals, including CGM data, lab results, and lifestyle information. This allows the DT system to generate precise, real-time recommendations for dietary and lifestyle modifications based on the predicted PPGRs of individual patients.

In contrast, Livongo focuses on providing remote monitoring and telehealth coaching, with device integration aimed at promoting patient self-management. Livongo’s system connects to various devices but relies exclusively on self-monitoring of blood glucose (SMBG) data, focusing on behavioral support rather than creating dynamic patient-specific models using CGM (29). Similarly, Omada Health targets diabetes prevention rather than management by offering structured lifestyle intervention programs that emphasize behavior modification, weight loss, and exercise, but it lacks the predictive modeling capabilities essential for personalized diabetes management (30). One Drop, on the other hand, uses AI-based analytics to offer basic glucose forecasts and insights derived from self-reported data. It does not have the capability to incorporate continuous biosignals or provide real-time recommendations (31). Meanwhile, mySugr is primarily a tracking and logging app that offers simple analytics based on manual inputs, making it less sophisticated than the DT system in terms of real-time adaptive capabilities (32).

When comparing predictive capabilities, the DT technology excels due to its use of advanced machine learning models like Gradient Boosting and LSTM networks. These models can accurately forecast glucose levels and provide adaptive recommendations based on ongoing CGM inputs. Other platforms, like Livongo (29) and Omada (30), rely mainly on historical data trends and static analytics, resulting in generic, non-dynamic suggestions. Even One Drop’s predictive glucose modeling is limited, as it cannot fully utilize biosignals or generate real-time personalized dietary recommendations (31). The DT system’s ability to integrate multiple data streams (e.g., CGM, lab tests, medication, and sleep patterns) into a unified digital model stands in contrast to the siloed approach of most other platforms, which either rely on isolated device data or focus exclusively on a single aspect of diabetes management.

In terms of clinical outcomes, the DT technology achieved a 2.9% reduction in HbA1c, with 94% of participants discontinuing T2D medications and 72.7% achieving remission at 1 year through a real-time personalized management approach (18). In comparison, Livongo reported a 1.1% reduction in HbA1c at 6 months (29) and One Drop achieved a 1.36% HbA1c reduction at a median follow-up of approximately 4 months (31), both primarily through behavioral coaching. mySugr observed improvements in mean blood glucose (−10.4%), tests in range (+8.5%), and estimated HbA1c (eA1c) (−0.4%) over two months by enhancing adherence to self-monitoring practices (32). Omada Health’s outcomes are more centered on diabetes prevention, focusing on weight reduction and lowering the risk of developing T2D (30).

User experience is another differentiating factor. The DT system offers a highly personalized, adaptive interface with continuous monitoring and real-time actionable insights. Livongo and Omada Health, offer straightforward, user-friendly interfaces but with less customization and flexibility (29, 30). One Drop’s interface is intuitive but relies heavily on user input, making it less ideal for patients seeking automated support (31). mySugr provides a simplified tracking and logging interface, which is suitable for basic data recording but lacks advanced features for precision care (32). The key advantage of the Digital Twin technology is its ability to create a dynamic, patient-specific digital model for real-time, precise interventions, unlike Livongo’s focus on telehealth coaching (29), Omada Health’s structured lifestyle programs (30), One Drop’s AI-driven insights (31), and mySugr’s simple tracking features (32).

The DT technology is an advanced tool for individualized diabetes management, offering real-time predictive modeling, comprehensive data integration, and personalized recommendations. Unlike generic platforms, it builds a unique digital replica for each patient, capturing detailed responses to diet, medication, and exercise. Using machine learning models such as Gradient Boosting and LSTM, the DT system predicts PPGRs and adapts recommendations based on real-time CGM data. It incorporates diverse inputs—lab tests, medications, sleep, and activity—providing a more complete health profile than other platforms that rely on limited or self-reported data. Its real-time, meal-specific guidance makes it superior to static dietary recommendations, supporting highly tailored and dynamic diabetes management.

### Advantages

The DT technology offers several distinct advantages over conventional approaches, particularly in its ability to deliver precision and personalization through the use of AI and machine learning. By accounting for each patient’s unique metabolic responses, the DT enables more effective interventions and improved clinical outcomes. Its digital nature also enhances scalability and accessibility, with the DT app and connected devices providing an intuitive interface that encourages patient engagement and adherence. Furthermore, the DT technology’s comprehensive approach extends beyond glycemic control, offering benefits across various health domains, including weight management, cardiovascular health, and mental well-being. Real-time monitoring and data integration allow for dynamic adjustments to recommendations, ensuring that treatment remains effective as the patient’s condition evolves.

### Limitations

The success of the DT technology relies heavily on accurate and complete data input, which can be impacted by language barriers, user proficiency, and patient compliance with food logging and device usage. Incomplete tracking of meals, activity, or health metrics, along with technical issues like calibration errors in wearables or CGMs, may affect the accuracy of recommendations. Additionally, its generalizability to diverse populations with varying dietary habits, socioeconomic conditions, and access to technology is a concern. The regulatory landscape poses another challenge, as evolving data privacy laws like HIPAA and GDPR may require continuous adaptation for compliance. Self-reported adherence to app-based interventions may also introduce bias in tracking engagement and outcomes.

User engagement, dietary compliance, and sustained behavior change can be challenging due to fluctuating motivation, stress, competing priorities, and psychological barriers like decision fatigue, habit resistance, and emotional eating. App fatigue and declining interest may also affect long-term adherence. The DT platform addresses these challenges through dynamic AI-generated nudges, delivered via the mobile app, providing personalized, real-time feedback based on data from CGM and activity trackers. Machine learning models continuously process this data to recommend tailored adjustments to diet, physical activity, and sleep, guiding users toward healthier choices. These adaptive nudges promote engagement, reinforce adherence, and support sustainable behavior changes over time.

Despite these limitations, the AI-powered DT technology effectively mitigates many issues through continuous learning from real-time data, allowing the system to adapt to patient behavior and compensate for missing or inaccurate inputs. Predictive analytics and enhanced user interfaces, including multilingual support, improve compliance and reduce communication barriers. The system’s self-calibration feature ensures accurate performance by minimizing errors from device malfunctions. Additionally, the platform’s flexible design accommodates varying dietary habits, socioeconomic conditions, and levels of digital literacy, promoting sustained engagement and long-term success.

To accommodate diverse dietary patterns, the DT technology incorporates local food preferences into meal plans, optimizing adherence and outcomes. For varying socioeconomic conditions, cost-effective food and exercise options are recommended, along with social support mechanisms to enhance adherence. To address varying levels of digital literacy, the app features a simplified design and personalized guidance, ensuring broad accessibility and making the intervention effective for a wider audience.

### Future directions

To improve the DT technology, future research should focus on expanding the dataset to include more diverse populations, enhancing generalizability across ethnic, age, and socioeconomic groups. Incorporating additional health metrics like genetic or microbiome data could further personalize recommendations and improve outcomes.

Assessing the long-term sustainability of DT’s benefits, particularly in maintaining T2D remission and preventing relapse, is crucial. Longitudinal studies should explore the lasting effects on clinical outcomes, such as sustained remission, cardiovascular health, and complication prevention. Enhancing participant adherence through user-friendly interfaces, personalized feedback, and real-time support will also increase platform effectiveness.

Further refinement of machine learning algorithms is essential, especially by integrating more complex data for greater predictive accuracy. Optimizing the app’s nudge system using behavioral science techniques can reduce alert fatigue and encourage lasting behavior change.

As regulatory frameworks evolve, collaboration with policymakers is key to ensuring compliance and adaptability. Pharmacoeconomic analyses comparing the DT technology to traditional T2D management could demonstrate its cost-effectiveness, aiding decision-making for healthcare providers and policymakers.

## Conclusion

The DT technology represents a transformative approach to the personalized management of T2D. Integrating AI, ML, and real-time data monitoring, the DT technology offers a highly effective method for improving glycemic control, achieving T2D remission, and enhancing overall metabolic health. While there are challenges related to data accuracy, patient compliance, and device reliability, the DT technology’s advantages far outweigh these limitations. The results achieved in this study underscore the potential of precision medicine to revolutionize T2D care and provide a pathway to sustained remission for a significant proportion of patients.

## Data Availability

Publicly available datasets were analyzed in this study. This data can be found here: Data will be available on reasonable request from the corresponding author.
